# Could the Plague Attack England?

**Published:** 1896-12-19

**Authors:** 


The Hospital

A Journal of

The Medical Sciences and Hospital Administration,

Vol. XXI.—No. 534.] [Dec. 19, 1896.

### Could the Plague Attack England?

It is only a little more than two hundred years since London suffered from a terrible epidemic which carried off about 70,000 people, and is known in history as the Great Plague of London. For three hundred years before that event plague had been more or less prevalent in various parts of Europe, and had over and over again taken an epidemic form and caused great loss of life. During the earlier outbreaks the destruction was something frightful to contemplate. It has been calculated by Hecker that no less than twenty-five million persons, or one-fourth of the whole population of Europe, died in the epidemics which swept across the land in the middle of the fourteenth century, and in certain parts of England it would appear that the whole population was carried off. In the fifteenth, in the sixteenth, and in the seventeenth centuries successive epidemics recurred; but since then England has been free. The disease receded eastwards, most of the countries in Europe becoming clear of it towards the end of the seventeenth century; Poland, Russia, and the Danubian countries, however, remained affected till considerably later. But the disease seems always to have persisted in one part or another of that vague geographical expression—the East, and has again and again stretched out its tentacles to gather victims in Turkey, in Russia, and along the shores of the Mediterranean.

At last, about 1845, the shores of the Mediterranean became free, not, however, from the disease vanishing, but merely from it retiring still more to the east, into the mountainous district of Kurdestan. Plague has neither died away nor changed its character. In 1853 there was an outbreak in Arabia, and others have occurred again and again in the same part, the last taking place so lately as 1889.

In an outbreak on the Volga in 1878, as in the old "black death," whole villages were destroyed, the general mortality being nearly 90 per cent. of the attacks. In China, plague has probably been endemic for a long time, but, as in all other countries, its epidemic virulence has varied greatly from time to time. In 1894, however, it broke out again in Canton, and was quickly carried to Hong Kong, and now—as everybody knows—we have it in Bombay, and probably Calcutta.

Whether the suspicious cases that have recently occurred in London, and have been investigated by the Local Government Board, have been cases of plague or not, it at least becomes a matter of no small interest to inquire how far England may consider herself safe from the ravages of this terrible disease. It is one of the commonplaces of history, taught to all school children, that the great Fire of London was a blessing in disguise, and so purified London that we have remained free from plague ever since. This, however, is a very one-sided and imperfect view of the situation. Plague existed in plenty of other places which were not burned down, and plague gradually retired across a whole continent, apparently quite irrespective of any changes in the customs of the inhabitants, or any modification of their sanitary surroundings. No doubt the sanitary conditions in which many of the people live have been vastly improved since the time of the black death of the fourteenth century. But both then and in the time of the Great Plague many smug and comfortable citizens were carried off, so that it was not altogether a matter of poverty; and, as to sanitation, it would, we think, be idle to contend that towards the end of the seventeenth century the sanitary surroundings became so much better, the poverty so much less, and the food so much more ample throughout Europe than they had been during the three preceding centuries, that the plague, which up to then had occurred so frequently, could no longer find a foothold among the people.

With sadness we have to accept the fact that we do not know on what depends the periodical development of epidemic virulence in plague. We know that plague remains endemic in certain districts in a minor form for long periods, and then breaks bounds, spreading far and wide as an intensely fatal epidemic, but why this happens we do not know. We know that, whether in regard to endemic prevalence or epidemic spread, the presence of filth, and especially of pollution of the soil, seems to be a determining agent in regard to the localities to be attacked. But in view of the fact that plague vanished of its own accord from Europe during the seventeenth century, it would be presumptuous to say that the improvement which has taken place in such conditions will prevent its recurrence. So far as our sanitation helps to protect us from other zymotic diseases, so it will do from plague, for plague is essentially a filth disease. But sanitation is a wide term. We are told that overcrowding of dwelling houses is a powerful contributory cause of plague; but have we no overcrowding? We are told that "of all social conditions, poverty and general social misery seem to be the most influential in its production" (*Allbutt's System of Medicine*). But have we no poverty and social misery? And as for dirt and pollution of the soil, although our well-drained towns may probably assume that they are safe from a disease so peculiarly allied with filth as plague is, we would ask: Are there no midden towns in England? Are there no villages yet undrained? Are there no hamlets yet unprovided with water except such as soaks into the wells from neighbouring cesspools? And we would ask doubters to peruse some of the reports of the medical officer to the Local Government Board on such subjects. We do not wish to be alarmist, but to those who boast that England is immune to such diseases, and that by her cleanliness she is saved, we say: Do not boast too soon.

